# Printed by Parkinson’s: a neurological art project linking patient stories and biosignals

**DOI:** 10.1186/s42466-020-00084-y

**Published:** 2020-11-04

**Authors:** Lucia K. Feldmann, Andrea A. Kühn

**Affiliations:** 1 Department of Neurology, Charité Universitätsmedizin Berlin, corporate member of Freie Universität Berlin, Humboldt-Universität zu Berlin, and Berlin Institute of Health, Charitéplatz 1, 10117 Berlin, Germany; 2 Berlin School of Mind and Brain, Humboldt-Universität zu Berlin, Berlin, Germany; 3 Deutsches Zentrum für Neurodegenerative Erkrankungen, Berlin, Germany; 4 NeuroCure Clinical Research Centre, Charité Universitätsmedizin Berlin, Berlin, Germany

**Keywords:** Patient outreach, Parkinson’s disease, Art

## Abstract

“Printed by Parkinson’s” is an innovative project with the main aim to raise awareness for the many aspects of Parkinson’s disease and their implication for everyday life. In a cooperation of Innocean Worldwide GmbH and the Movement Disorder and Neuromodulation Section, Charité Universitätsmedizin Berlin, design and medical and neuroscientific expertise were combined to create unique artworks: Bronze sculptures were created when combining personal objects selected by each patient, and their neurophysiological individual health data. As a core element, patient interviews in an accompanying film shed light on the personal stories behind the art objects. Public presentations raised interest in the topic and very positive reactions by patients and relatives, and we think that the possibility to use art for improved communication in the field of medicine holds promise for the future.

## Introduction

Parkinson’s disease (PD) is a condition which changes the patients’ lives in many aspects. While PD is a routine diagnosis for neurologists, for patients, the diagnosis may initially raise fears and negative images of disability and decay. Coping with the various motor and non-motor symptoms is a long process accompanied by many visits to the hospital and continuous therapy adjustments. Medical therapies themselves may be demanding, with side-effects such as impulse control disorders and severe fluctuations dictating the daily life. However, taking the decision for advanced invasive therapies such as deep brain stimulation is often a long process for each patient and his family. The impact of PD on the emotional and social life – for the patients and their relatives – is often underestimated.

Patients and their histories are an incredibly valuable resource for helping PD patients and their families in the many stages of the progressing disease. One important way to use this resource are support groups, but here, we took a different approach: In cooperation with an international communications company [2], we developed an arts project for which sculptures were created by merging neuroscience and design. Very personal patient stories were captured in compelling interviews about the PD patients’ lives with the disease, with the aim to raise awareness for the many layers of this common neurological disorder.

## Making-off “Printed by Parkinson’s”

**Fig. 1:**
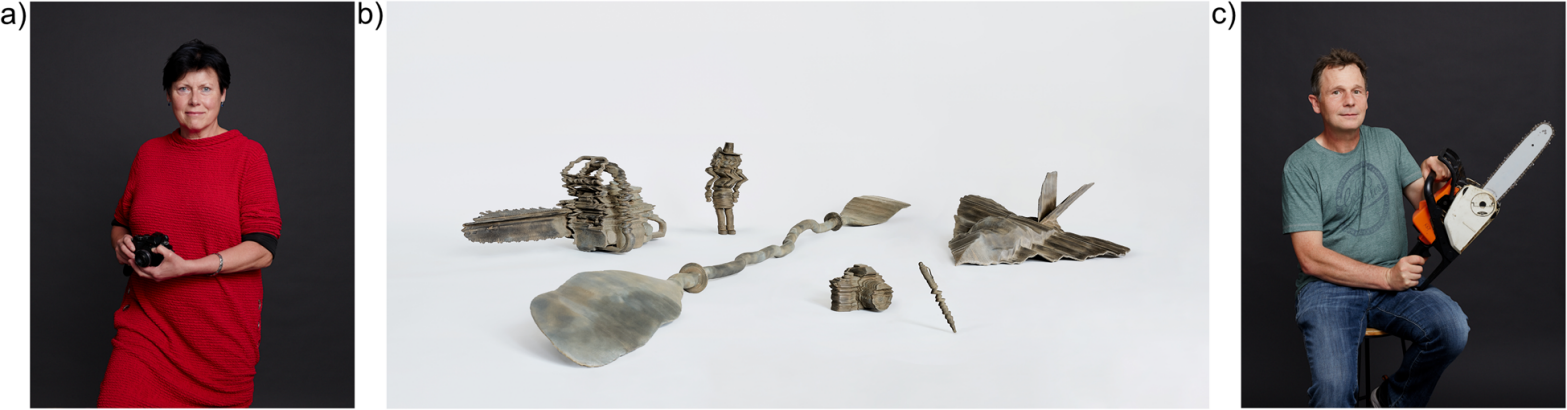
Printed by Parkinson’s – design and patients’ personal stories. Patients selected different objects representing their experience with PD: **a** a camera symbolizes difficulties and reacquisition of creative expression for this patient; **c** for the passionate former motorcyclist, using the chainsaw for handicraft and gardening is a favorite occupation beside rock-climbing, which he even started after the diagnosis. Individual electrophysiological data and selected objects were used to create unique bronze sculptures (**b**). Photographs were taken by Ender Suenni (photographer, Cosmopola GmbH)

“Printed by Parkinson’s” was an intensive and exciting project bringing together creative minds in design, film, photography, neuroscience, medicine and, importantly, the patients. Six PD patients from our out-patient clinic were asked to name an object that had become difficult to use due to PD and had a personal meaning to them. The result was an interesting variety (Fig. 1a+c): while one patient selected a camera which had helped her continue with her creativity, another patient selected a nutcracker which he had loved to build but was now struggling to construct with limited fine-motor skills. One patient chose a paddle, which he connected to PD because during medication induced impulse control problems he had ended up in distress, unable to navigate on the open sea.

For the project, we selected patients who had participated in electrophysiological recordings. All patients had received DBS and had participated in local field potential (LFP) recordings for clinical studies and research projects during the period of externalization in the two-staged DBS surgery. During this period, we used the opportunity to record LFPs from the deep brain target structures for DBS, in the case of PD, the subthalamic nucleus (STN). Research using these unique recordings has previously contributed to a better understanding of the pathophysiology underlying movement disorders [3, 4, 6, 7]. Another data source were movement recordings using an accelerometer. Here, we re-used these patient data in order to create individual art objects reflecting the personal pathological activity and symptomatology. Specifically, a 3-D-printer was “inflicted” with the disease using the patient’s individual electrophysiological data thereby creating individualized art objects [5]. The result are mesmerizing bronze sculptures from the objects that the patients had chosen (Fig. 1b).

The core element of “Printed by Parkinson’s” is the film, which mainly consists of very personal interviews with the patients, as well as an interview with the head of the Movement Disorder Unit, Prof. Dr. Andrea Kühn. This film gives the patients the opportunity to tell their stories, their fears and struggles, their strategies for coping and how they won back their lives after the initial shock of the diagnosis. Together with clinical background information, this film has the potential to reach patients and their relatives on a more personal level and convey understanding, hope and support. The film can be viewed on the project website https://www.printedbyparkinsons.com and on the homepage of the Movement Disorder and Neuromodulation Unit, Department of Neurology, Charité Berlin.

## Reception and outlook

The film and the sculptures were first displayed publicly on World Brain Day at a vernissage and exhibition in the Alte Münze, a renowned museum space in the center of Berlin. It was furthermore presented during support group and charity events. Art prints and sculptures are now on display at the department of Neurology, Charité, Berlin. While it was also a success in the field of design winning several prizes [1, 8], our true reward was the very positive feedback by patients and relatives. We hope that “Printed by Parkinson’s” will continue to raise awareness for the personal side of PD at other exhibitions, e.g. the new Humboldt Lab in the Humboldt Forum in Berlin, and will motivate and support our PD patients. Through the combination of arts and neuroscience, we hope to multimodally achieve understanding and support for PD and other movement disorders.

## Data Availability

Not applicable.

## Abbreviations

PD: Parkinson’s disease
DBS: Deep brain stimulation
LFP: Local field potential
STN: Nucleus subthalamicus
